# A pharmacokinetic–pharmacodynamic model for chemoprotective agents against malaria

**DOI:** 10.1002/psp4.12875

**Published:** 2022-11-22

**Authors:** Mohammed H. Cherkaoui‐Rbati, Nicole Andenmatten, Lydia Burgert, Oluwaseun F. Egbelowo, Rolf Fendel, Chiara Fornari, Michael Gabel, John Ward, Jörg J. Möhrle, Nathalie Gobeau

**Affiliations:** ^1^ Medicines for Malaria Venture Geneva Switzerland; ^2^ University of Basel Basel Switzerland; ^3^ Division of Clinical Pharmacology, Department of Medicine The University of Texas at Austin Texas Austin USA; ^4^ Institute for Tropical Medicine University of Tübingen Tübingen Germany; ^5^ AstraZeneca Cambridge UK; ^6^ Center for Modelling and Simulation in the Biosciences, BioQuant‐Center University of Heidelberg Heidelberg Germany; ^7^ Department of Mathematical Sciences University of Loughborough Loughborough UK; ^8^ Present address: Pharmaceutical Sciences, Pharma Research and Early Development Hoffmann‐La Roche Basel Switzerland; ^9^ Present address: Lonza AG Visp Switzerland

## Abstract

Chemoprophylactics are a vital tool in the fight against malaria. They can be used to protect populations at risk, such as children younger than the age of 5 in areas of seasonal malaria transmission or pregnant women. Currently approved chemoprophylactics all present challenges. There are either concerns about unacceptable adverse effects such as neuropsychiatric sequalae (mefloquine), risks of hemolysis in patients with G6PD deficiency (8‐aminoquinolines such as tafenoquine), or cost and daily dosing (atovaquone–proguanil). Therefore, there is a need to develop new chemoprophylactic agents to provide more affordable therapies with better compliance through improving properties such as pharmacokinetics to allow weekly, preferably monthly, dosing. Here we present a pharmacokinetic–pharmacodynamic (PKPD) model constructed using DSM265 (a dihydroorotate dehydrogenase inhibitor with activity against the liver schizonts of malaria, therefore, a prophylaxis candidate). The PKPD model mimics the parasite lifecycle by describing parasite dynamics and drug activity during the liver and blood stages. A major challenge is the estimation of model parameters, as only blood‐stage parasites can be observed once they have reached a threshold. By combining qualitative and quantitative knowledge about the parasite from various sources, it has been shown that it is possible to infer information about liver‐stage growth and its initial infection level. Furthermore, by integrating clinical data, the killing effect of the drug on liver‐ and blood‐stage parasites can be included in the PKPD model, and a clinical outcome can be predicted. Despite multiple challenges, the presented model has the potential to help translation from preclinical to late development for new chemoprophylactic candidates.

**Study Highlights**

WHAT IS THE CURRENT KNOWLEDGE ON THE TOPIC?

The administration of chemoprophylactic medicines to healthy subjects to prevent them from developing malaria is a key strategy in protecting populations at high risk. However, current antimalarial drugs aimed at protecting people through chemoprophylaxis often come with concerning adverse effects, are expensive, or require courses of daily dosing. There is thus a need to discover and develop new chemoprophylactic drug candidates to provide more affordable therapies with better adherence. To effectively discover and develop chemoprophylactic medicines requires knowledge of how they kill or otherwise inhibit parasites in both the liver and blood stages of the lifecycle. Although data are available from induced blood‐stage malaria volunteer infection clinical studies and, separately, sporozoite‐infected human challenge studies, there is no known published work on the integration of the two to create a model for both the liver and blood stages of *Plasmodium falciparum* growth.

WHAT QUESTION DID THIS STUDY ADDRESS?

Is it possible to make inferences about parasite levels and activity in the liver stage of the malaria infection process, which cannot be measured directly, by mathematically modeling the pharmacokinetic and pharmacodynamic relationships between chemoprophylactic drugs, such as DSM265 and blood‐stage parasites, thereby increasing our ability to predict clinical outcomes and thus improving drug development?

WHAT DOES THIS STUDY ADD TO OUR KNOWLEDGE?

The study shows that by combining qualitative and quantitative knowledge of parasite dynamics from two different study designs, induced blood‐stage malaria and sporozoite‐infected human challenge studies, to create a mathematically realistic pharmacokinetic–pharmacodynamic model of malaria parasite and chemoprophylactic drug interaction, it was possible to infer information about liver‐stage parasite levels and activity. By integrating the two designs of study, it was also shown that it is possible for the model to predict clinical outcomes. By assuming similar characteristics between the liver‐ and blood‐stage activities, it was possible to predict the level of chemoprotection afforded by DSM265.

HOW MIGHT THIS CHANGE DRUG DISCOVERY, DEVELOPMENT, AND/OR THERAPEUTICS?

One of the biggest challenges facing malaria treatment and prevention is a problem of knowledge concerning liver activity during the malaria parasite infection process. However, the ability to infer such information by way of mathematical modeling is a means of changing this. If blood‐stage activity is known, then pharmacokinetic–pharmacodynamic models can be used to assess the level of chemoprotection delivered by compounds with similar liver‐ and blood‐stage activities in in vitro systems. This could be particularly useful in candidate selection for phase III clinical trials if such data were combined with knowledge from epidemiology and “adherence behavior,” augmenting the transition from preclinical to the latter stages of development for new and improved antimalarials.

## INTRODUCTION

Despite an unprecedented period of success throughout the 21st century, malaria remains a significant global health challenge that in 2019 killed 409,000 people worldwide, of which 67% were children younger than the age of 5.^1^ Although fatalities from malaria are falling, cases remain stable, and current measures are unable to prevent initial infection. In addition to children aged younger than 5, pregnant women and nonimmune adults are particularly susceptible.^2^ With no fully protective vaccine and the constant threat of insecticide resistance, new drugs to prevent infection are needed.

Administration of chemoprophylactics to healthy subjects to prevent malaria development is a key tool in eradication. Some 25 million children aged younger than 5 are protected each season using a monthly treatment dose of sulfadoxine–pyrimethamine plus amodiaquine costing around $1 per child‐year for the drug and $5 in program costs.^3^ Furthermore, there may be a promising alternative with dihydroartemisinin–piperaquine.^4^ However, there are concerns that resistance to these active drugs will lead to these regimens being compromised. There are other options available for prophylaxis. Some come with concerning adverse effects: mefloquine is associated with neuropsychiatric events and tafenoquine with hemolysis in G6PD‐deficient subjects, and both require weekly dosing. Atovaquone–proguanil is expensive to manufacture, and doxycycline cannot be administered to children; both require daily dosing.^2^, ^5^ Combined with the development of resistance to older antimalarial prophylactics (e.g., chloroquine or artemisinin combination therapies), new chemoprophylactic agents are needed. These would provide more affordable therapies and pharmacokinetic (PK) properties that support and allow weekly or monthly dosing.^6^

Development of chemoprophylactic molecules requires understanding of activity on all parasite life stages. After a mosquito bite, sporozoites travel to the liver and infect hepatocytes, where they multiply and mature into schizonts for 5–7 days^7^; infected hepatocytes then rupture, and merozoites are released into the bloodstream where they infect erythrocytes. The parasite then undergoes a cycle of asexual multiplication (e.g., 48‐h cycle for *Plasmodium falciparum*) with rupturing of erythrocytes and reinfection. It is the periodic rupture at this stage that causes malaria clinical symptoms. To prevent malaria infection, molecules can either have causal prophylaxis, stopping the development of parasites in the liver (e.g., atovaquone–proguanil, tafenoquine, or pyrimethamine), or suppressive prophylaxis, inhibiting the development of parasitemia (parasite levels in blood; e.g., mefloquine).

To prioritize new medicines, it is important to have a validated model of infection in humans that combines these two aspects, whereby predictions can be made regarding parasitemia supporting the development of suppressive prophylaxis, but also predictions on the prophylactic efficacy based on inferred liver activity. To help build such a model, data from DSM265, a new compound that selectively inhibits parasite dihydroorotate dehydrogenase (DHODH), was used. It shows in vitro activity against the liver and blood stages of *P. falciparum* ^8^ and has a long elimination half‐life in humans of around 96 h, which could support once‐weekly therapy.^9^ The potential of DSM265 as a treatment and chemoprophylactic was assessed in two different types of volunteer infection study (VIS), namely, an induced blood‐stage malaria (IBSM) using intravenously administered infected erythrocytes and an intravenous (i.v.) delivery of sporozoites in a human challenge model (spz HuCh).

In IBSM studies, *P. falciparum*–infected erythrocytes were administered intravenously to healthy subjects. Once parasitemia reached a predefined threshold but was still below the clinical symptom threshold, DSM265 was administered. Parasitemia was monitored daily from infection to 1 month after dosing.^9^ Clearance of parasites due to treatment could be observed, as could potential recrudescence if the dose was subtherapeutic. Different doses were usually tested to characterize pharmacokinetic–pharmacodynamic (PKPD) relationships of the drug on blood‐stage parasites.

In spz HuCh, sporozoites were either injected intravenously or administered via mosquito bites.^10^, ^11^ This reflects the natural infection of *P. falciparum*, where parasites first invade the liver and later appear in blood. The drug to be tested was administered prior to infection, usually at one‐dose level with different time intervals between administration and infection, or different dose levels with a fixed time interval. Parasite levels in the liver cannot be directly measured, hence only parasite levels in blood were monitored. Parasitemia was initially below a limit of quantification (BLOQ) and became measurable if the administered drug dose was insufficient to kill either liver‐ or blood‐stage parasites. Using these studies, the presented PKPD model was developed to semimechanistically describe parasite dynamics in two compartments, the liver and blood, where transfer occurs only at maturation of merozoites at Day 6. Analogous to analyzing oral and i.v. PK by use of an absorption compartment with a lag time, the model deconvolutes drug activity for the unobserved liver stage to better capture the ability of DSM265 to provide protection.

## MATERIALS AND METHODS

### Ethics disclaimer

Data for this PKPD analysis were taken from existing publications. All participants gave written informed consent before being included in studies; all were approved by their institutional review boards.

### Data

DSM265 dried blood spot (DBS) concentrations and parasitemia data were pooled from 15 different studies. These consisted of one first‐in‐human (FIH) study, two IBSM studies, one phase IIa study, two spz HuCh studies, and nine published studies to obtain control data from nontreated subjects. The FIH study was a PK single‐ascending‐dose study where DSM265 was administered to healthy volunteers; each subject provided a rich PK sampling profile.^9^ The two IBSM studies examined healthy volunteers who were inoculated with *P. falciparum*–infected erythrocytes and treated with DSM265 7 days after inoculation; PK and parasitemia were monitored in each subject. Parasitemia was measured prior to drug administration, providing information on the natural net growth of parasite in the blood stage. The phase IIa study was conducted in Peru with uncomplicated malaria patients (blood stage) who were treated with DSM265 at the time of diagnosis; both DSM265 concentrations and parasitemia were monitored.^12^ The two spz HuCh studies evaluated 400‐mg DSM265 1, 3, and 7 days before i.v. injection of sporozoites; two placebo individuals were assigned to each cohort.^10^ In addition, data from a further nine studies were extracted from Coffeng et al.^13^ These observed healthy volunteers were placebo subjects in trials of other drugs and were inoculated intravenously with sporozoites. This allowed the development of malaria from liver‐stage infection to be described.

### PK modeling

DSM265 has been described by McCarthy et al.^9^ with a two‐compartmental model including zero‐order and dose‐dependent duration of absorption and bioavailability. This model was used as a starting point where a lag time in absorption was also assessed. Dose level was tested as a covariate on the duration of absorption and bioavailability instead of estimating different values for different doses. PK characteristics were evaluated with nonlinear mixed‐effects modeling using the software Monolix (Version 2018R2). Individual subject PK parameters were carried forward into the PKPD model as regressors to derive PD parameters for blood‐ and liver‐stage activities.

### Chemoprophylaxis PKPD model

**FIGURE 1:**
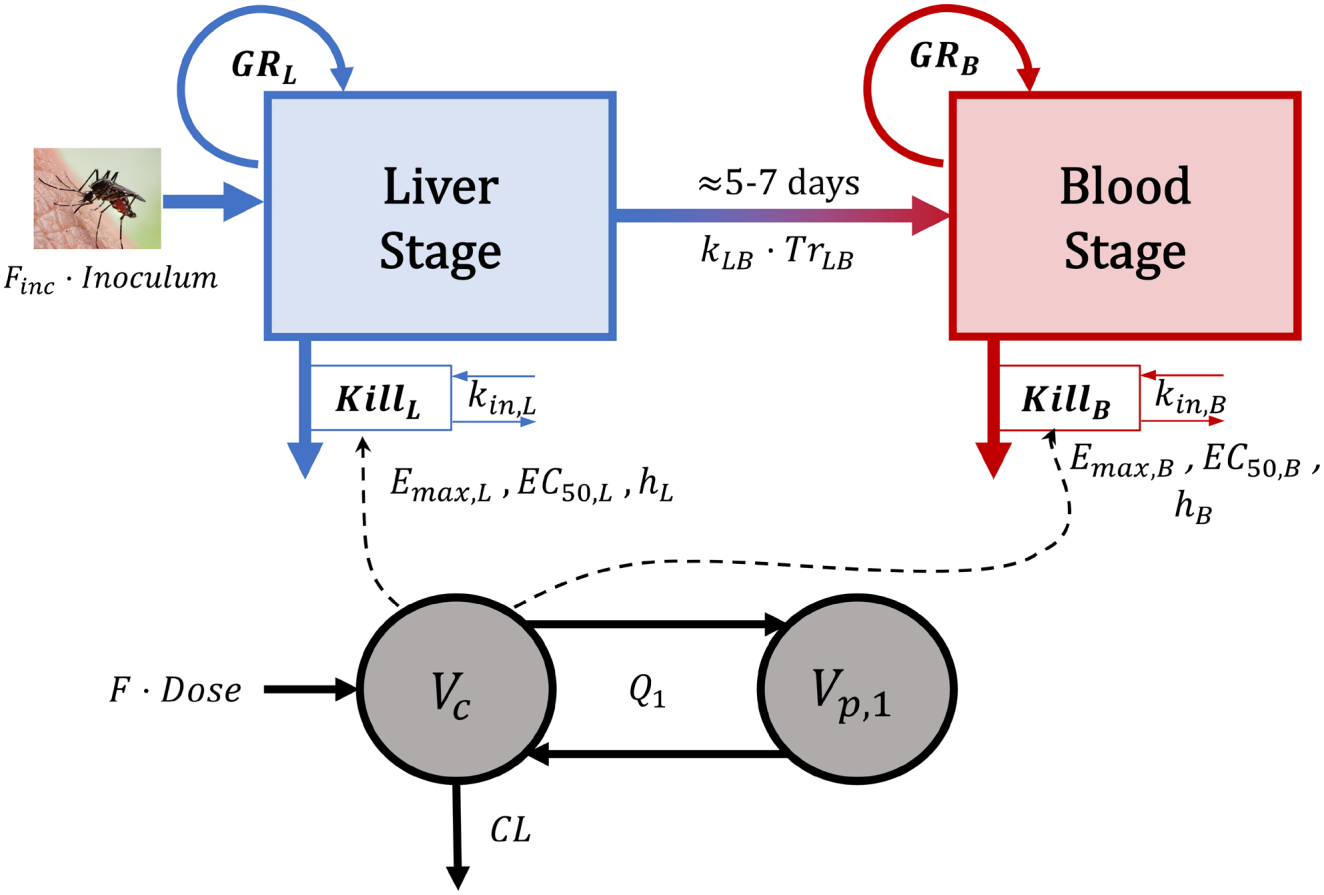
Schematic representation of DSM265 pharmacokinetic and the two‐stage pharmacodynamic model for *Plasmodium falciparum*. CL, clearance; $E_{max,X}$, maximum killing rate; $EC_{50,X}$, dried blood spot concentration at which half of maximum effect is attained; F, bioavailability; $F_{inc}$, the fraction of infectious inoculated sporozoites that invade hepatocytes; $GR_{X}$, growth rate; $h_{X}$, Hill coefficient; where X represents B (blood) or L (liver); Inoculum, number of injected sporozoites; $k_{in,X}$, turnover rate; $k_{LB}$, rate of merozoites invading erythrocytes once they leave hepatocytes; $Q_{1}$, inter‐compartmental clearance; $Tr_{LB}$, cumulative fraction of hepatocytes that burst over time; $V_{c}$, volume of central compartment; $V_{p,1}$, volume of peripherical compartment.

A visualization of the chemoprophylaxis PKPD model is presented in Figure 1 with the set of coupled differential equations describing interplay between the liver and blood pools of parasites listed in the following:

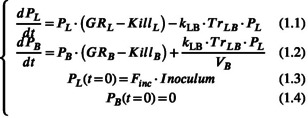




The PKPD model describes the dynamic of parasite number in the liver, $P_{L}$, and parasites per milliliter in blood, $P_{B}$, as the net balance of an exponential growth ($GR_{L}$, $GR_{B}$) and killing ( $Kill_{L}$, $Kill_{B}$) due to an antimalarial drug. The release of merozoites from the liver to blood is described by the term $k_{LB}\cdot Tr_{LB}\cdot P_{L}$, where $k_{LB}$ is the rate of merozoites invading erythrocytes once they leave hepatocytes, and $Tr_{LB}$ the cumulative fraction of hepatocytes that burst over time. $P_{B}$ being expressed in parasites per milliliter, the number of transferred merozoites is divided by $V_{B}$, the total volume of blood assumed to be the standard human value of 5 L for all individuals. $P_{L}$ was initialized at time 0 by Inoculum (the number of injected sporozoites) times $F_{inc}$, the fraction of inoculated sporozoites that invade hepatocytes; many sporozoites are nonviable or do not reach the liver.

$Kill_{L}$ and $Kill_{B}$ were described by a turnover model as DSM265, a DHODH inhibitor, leads through a cascade to inhibit DNA and RNA syntheses.^14^ The following equations were added to the system to introduce a lag time:

$$\begin{array}{c} \begin{array}{c} \left\{\begin{array}{cc} \frac{dKill_{L}}{dt}=k_{in,L}\cdot \left(\frac{E_{max,L}\cdot C_{\mathrm{DBS}}^{h_{L}}}{{\mathrm{EC}}_{\mathrm{50},L}^{h_{L}}+C_{\mathrm{DBS}}^{h_{L}}}-Kill_{L}\right) & \left(2.1\right)\\ \frac{dKill_{B}}{dt}=k_{in,B}\cdot \left(\frac{E_{max,B}\cdot C_{\mathrm{DBS}}^{h_{B}}}{{\mathrm{EC}}_{\mathrm{50},B}^{h_{B}}+C_{\mathrm{DBS}}^{h_{B}}}-Kill_{B}\right) & \left(2.2\right) \end{array}\right. \end{array} \end{array}$$

where $C_{DBS}$, $E_{max,X}$, $EC_{50,X}$, $h_{X}$, and $k_{in,X}$ are DSM265 DBS concentration, maximum killing rate, DBS concentration at which half of maximum effect is attained, the Hill coefficient, and the turnover rate, respectively.

Regarding release of merozoites, $k_{LB}$ was set to a fast value of six per hour (equivalent to a mean transfer time of 10 min, $1/k_{LB}$) as merozoites invade erythrocytes rapidly.^15^ $Tr_{LB}$ controlling time distribution of liver‐stage maturation was described by:

(3) $$Tr_{LB}\left(t\right)=\frac{1}{1+\exp\left(\frac{-\left(t-T_{50}\right)}{\sigma_{LB}}\right)}$$

where $T_{50}$ was the time by which half the hepatocytes burst after inoculation, and $\sigma_{LB}$ was the distribution of release around time $T_{50}$. Initially, $T_{50}$ was set to 6 days, as a mid‐value between 5 and 7 days,^7^ with $\sigma_{LB}$ at 0.1 h, thereby defining that more than 98% of merozoites were released between Day 6 −30 min and Day 6 +30 min.

### Blood‐stage modeling

Parasitemia data from the IBSM and phase IIa studies were used, where individual PK parameters were added as regressors in the PKPD relationship of DSM265 against *P. falciparum* in the blood stage. As infection started immediately in the blood, only the equation describing parasite dynamics in blood was used (Equations 1.2, 1.4, and 2.2). Identifiability of $h_{B}$ requires parasitemia minima to be observed that, here, was BLOQ. Therefore, to achieve stable convergence, $E_{max,B}$, $EC_{50,B}$ (including interindividual variability [IIV]), and $k_{in,B}$ were estimated with fixed values for $h_{B}$ between 1 and 10. Furthermore, as baseline parasitemia was different between the IBSM and phase IIa, study type was added as a covariate. The model with the smallest Akaike information criterion (AIC) was selected. As an indicator, minimum inhibitory concentration against blood stage, $MIC_{B}$, was estimated for each individual as:

(4) $$MIC_{B}=EC_{50,B}\cdot {\left(\frac{GR_{B}}{E_{max,B}-GR_{B}}\right)}^{\frac{1}{h_{B}}}$$

### Modeling growth of hepatic schizonts

Liver parasite growth is not directly observable in clinical studies, as this would require biopsies to estimate infected hepatocytes. These are not performed because of a low likelihood of detection due to a low number of ejected sporozoites by mosquito bite (upper limit ~1000^7^, ^16^); therefore, even lower infected hepatocytes number compared with liver cell mass (135 × 10^6^ cells/g of liver). The liver parasite growth rate was derived from biological knowledge. Each invaded hepatocyte releases 10,000 to 50,000 merozoites after the 5‐ to 7‐day liver stage.^7^, ^16^ Assuming exponential growth and using the mean value of 30,000 merozoites produced by each invaded hepatocyte after ~6 days, the growth rate in liver $GR_{L,\text{pop}}$ was calculated to be 0.072 per h. Only the initial number of invaded hepatocytes remained unknown, which is proportional to $F_{inc}$. Using placebo data from all spz HuCh studies, $F_{inc}$ was evaluated in two steps. In Step 1, the blood‐stage equations were used to estimate the blood parasite growth rate $GR_{B}$ for each individual from the isolated growth phase data. In Step 2, $F_{inc}$ and its IIV were estimated using the PKPD model, with $GR_{B}$ as a regressor from Step 1, fixing the liver parasite growth rate for each individual, $GR_{L,\text{indiv}}$, to be the population estimate $GR_{L,\text{pop}}$ times the ratio $GR_{B,\text{indiv}}/GR_{B,\mathrm{pop}}\;$ to maintain 100% correlation between the liver and blood growth rates relative to the population value. No distinction was made between volunteers who received five mosquito bites or an i.v. dose of 3200 sporozoites as the infection observed was similar. Therefore, each mosquito was assumed to deliver 640 sporozoites. As with the blood stage, minimum inhibitory concentration against liver stage, $MIC_{L}$, was estimated for each individual as:

(5) $$MIC_{L}=EC_{50,L}\cdot {\left(\frac{GR_{L}}{E_{max,L}-GR_{L}}\right)}^{\frac{1}{h_{L}}}$$

### Modeling drug activity against hepatic schizonts

Parasitemia data from the spz HuCh were used, excluding placebo, where individual PK parameters were added as regressors to characterize the PKPD relationship of DSM265 against *P. falciparum* in the liver stage. As DSM265 was administered prior to infection and may remain active in the blood stage after liver infection, the final chemoprophylaxis PKPD model was used to describe the data. DSM265 was shown to have liver activity in vitro and in vivo,^14^ and it is believed the mechanism of action is the same in both stages; therefore, $E_{max,L}$, $h_{L}$, and $k_{in,L}$ were fixed with no IIV as these were not identifiable with the available data to the population value of blood‐stage $E_{max,B}$, $h_{B}$, and $k_{in,B}$. Similarly, $GR_{L}$ and $EC_{50,L}$ were assumed to be 100% correlated to $GR_{B}$ and $EC_{50,B}$ with the same deviations from the population value within each individual. To reduce the number of parameters to estimate, only liver‐stage $EC_{50,L}$ and its IIV were estimated. The parameters $F_{inc}$, $GR_{L}$, and $GR_{B}$ and their IIV were fixed from previous steps.

### Model validation

Given that the sequential process used to build and assemble the final model could introduce bias due to different experimental individuals being used at each stage, validation was performed holistically by comparing observed and predicted success rates for each treatment group from the spz HuCh with DSM265. Observed success rates were estimated as the ratio of the number of malaria‐free volunteers at Day 28 (i.e., parasitemia below 20 p/ml, the polymerase chain reaction LOQ) after i.v. inoculations to the number of volunteers in each treatment group. The 90% confidence interval (CI) for each treatment group was calculated using the Clopper–Pearson interval^17^ by assuming that the protective effect was binomial. To estimate predicted success rates, 10,000 virtual trials with the same designs as the observed studies were simulated. For each virtual trial, PK parameters were sampled from the estimated individual PK parameters to increase the focus on PD predictability by minimizing population PK model misspecification; PD parameters were sampled from the population model accounting for IIV and uncertainty. Predicted success rates were then estimated for each virtual trial as the proportion of malaria‐free volunteers at Day 28. Medians and 90% CIs were estimated for each treatment group.

### Model assessment

To discriminate whether the final chemoprophylaxis model best described observed cure, the success rates were simulated as noted previously in two hypothetical scenarios for liver‐stage activity. The first scenario assumed that DSM265 was active only during the blood stage, that is, $E_{max,L}$ was zero; the second assumed that liver and blood activities were equivalent, that is, $E_{max}$, $EC_{50}$, h, and $k_{in}$ were the same. By comparing and contrasting the simulation predictions against the observed data, one can ascertain which scenario better described the cure success rates.

### Posology prediction

To evaluate the chemoprophylaxis PKPD model for predicting efficacy in a real‐world prophylactic paradigm, eight simplified scenarios were simulated. In all scenarios, infection occurred at Day 0 with the same level as in the spz HuCh studies (3200 sporozoites), with two doses of DSM265 at 400 mg administered weekly. The first dose was administered between Day −7 and Day 0 prior to infection. For each scenario, success rates and 90% CI at Day 28 after infection were estimated by sampling PK and PD parameters, accounting for IIV and uncertainty, for 100 virtual trials each with 100 patients.

### Software

All data processing, analysis, and modeling were conducted with R (Microsoft Open R 3.5.1) combined with the IQRtools package (Version 1.0.3, IntiQuan) and Monolix (Version 2018R2). Although there were few PK data points BLOQ, many parasitemia samples were BLOQ. Consequently, it was vital that the M3 method was implemented in Monolix to allow for categorical information from these samples to be included in the analyses. For sections of the analysis, for example, Step 5 estimation of $EC_{50,L}$, where 70%–80% of samples were BLOQ, the analysis was more categorical than traditional minimizing of residuals between predictions and measured observations. BLOQ data and predictions are visualized in the goodness‐of‐fit diagnostics supplement (Text S1).

## RESULTS

### Data

**FIGURE 2:**
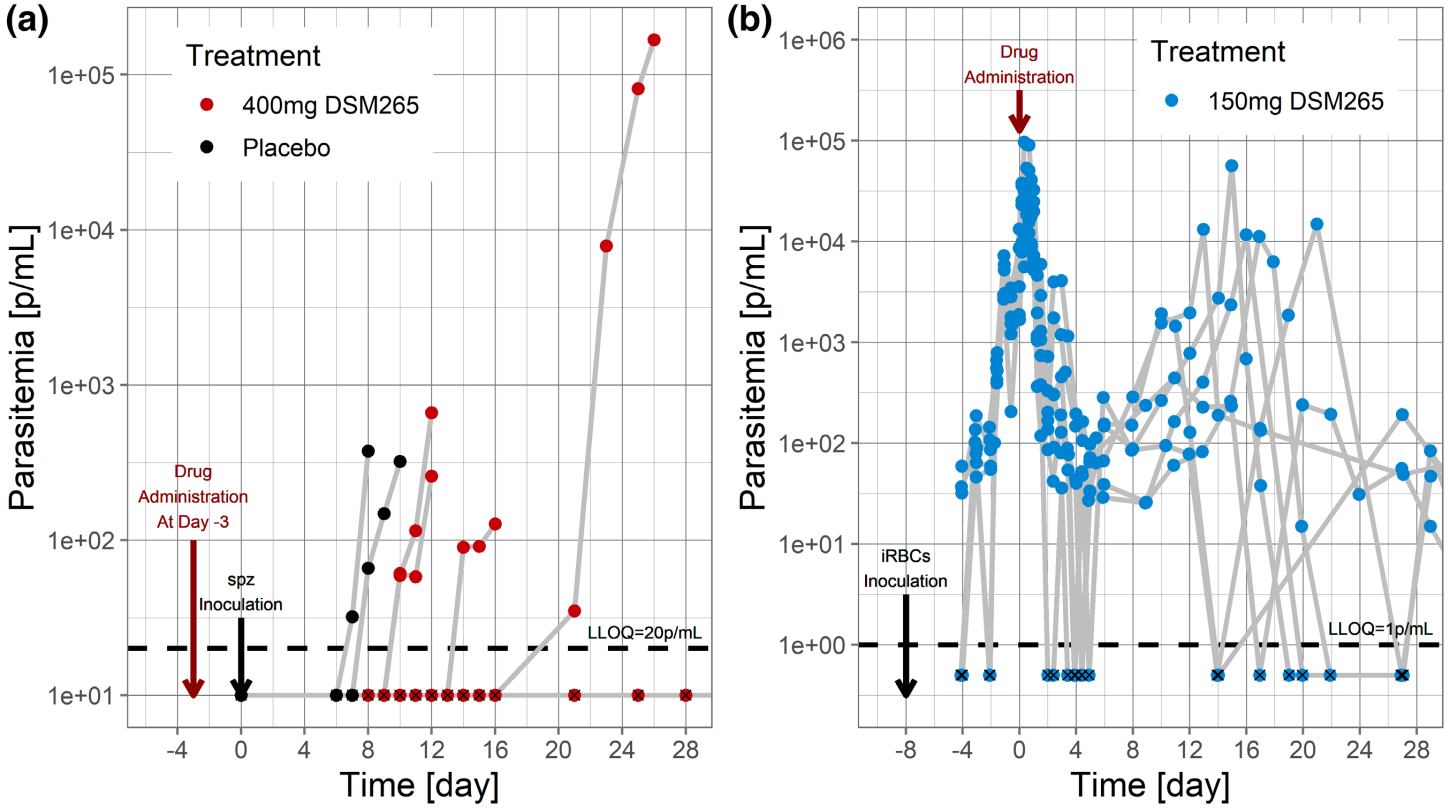
Example parasitemia data for (a) a placebo‐controlled sporozoite infection study investigating 400‐mg DSM265 and (b) an induced blood‐stage malaria study investigating 150‐mg DSM265. iRBC, infected red blood cell; LLOQ, lower limit of quantification; spz, sporozoite.

Data from 190 participants were compiled: 55 from FIH, 15 from IBSM, 24 from a phase IIa study of DSM265 treating uncomplicated blood‐stage infection, 39 from spz HuCh studies (including 10 placebo), and 57 placebos from published studies. Data are summarized in Table 1 and visualized in the goodness‐of‐fit supplement (Text S1), and Figure 2 illustrates the differences between a spz HuCh study and an IBSM study. Essential differences between the two study designs are worth considering. In a spz HuCh study, there are lag times before parasites are detected in blood, as parasite levels are not measurable in the liver, and these lag times differ between subjects. However, lags appear to be shorter in placebo trials as compared with those receiving the antimalarial drug. In an IBSM trial, all subjects show similar parasite growth curves prior to DSM265 administration, after which parasite numbers drop. If the dose was too low to kill all the parasites, then the number of parasites subsequently increased.

**TABLE 1:** Number of subjects per study type and treatment group

Treatment group	FIH	IBSM	spz HuCh	Phase IIa	Published	Total
Placebo	–	–	10	–	57	67
25‐mg DSM265	6	–	–	–	–	6
75‐mg DSM265	6	–	–	–	–	6
150‐mg DSM265	6	7	–	–	–	13
250‐mg DSM265	8	–	–	11	–	19
400‐mg DSM265	11	8	–	13	–	32
600‐mg DSM265	6	–	–	–	–	6
800‐mg DSM265	6	–	–	–	–	6
1200‐mg DSM265	6	–	–	–	–	6
400‐mg DSM265 (Day ‐1)^a^	–	–	5	–	–	5
400‐mg DSM265 (Day ‐3)^a^	–	–	6	–	–	6
400‐mg DSM265 (Day ‐7)^a^	–	–	18	–	–	18
Total	55	15	39	24	57	190
Reference citation	^9^	^9^	10, 11	^12^	^13^	

Abbreviations: FIH, first in human; IBSM, induced blood‐stage malaria; spz HuCh, sporozoites human challenge model.

^a^ Day prior sporozoite inoculation.

### PK model

PK of DSM265 was described by a two‐compartmental model with zero‐order absorption, a lag time, and linear elimination. IIV was estimated for all parameters except for inter‐compartmental clearance, $Q_{1}$, where IIV was set to zero due to high shrinkage. Regarding covariates, only dose level on relative bioavailability was statistically significant (reduction in AIC ≥10) and retained. The final PK model showed excellent overall diagnostic performance (Table 2; Text S1, Step 1).

**TABLE 2:** Population parameter estimates for the final model describing DSM265 pharmacokinetics in volunteers and patients with uncomplicated *Plasmodium falciparum* malaria (Text S1, Step 1)

Parameter	Unit	Value	IIV^a^	CV^b^	Distribution
*Typical parameters*					
F	–	1 (FIX)	0.209 (11.2%)	21.1%	Log‐normal
CL	L/h	0.476 (2.71%)	0.249 (8.4%)	25.3%	Log‐normal
$V_{c}$	L	8.58 (12.8%)	1.06 (10.2%)	144%	Log‐normal
$Q_{1}$	L/h	37.1 (4.61%)	0 (FIX)	0.0%	Log‐normal
$V_{p,1}$	L	57.3 (3.36%)	0.281 (8.75%)	28.7%	Log‐normal
$T_{k,0}$	h	2.85 (5.84%)	0.563 (7.43%)	61.1%	Log‐normal
$T_{lag,1}$	h	0.147 (12.7%)	0.72 (12.7%)	82.4%	Log‐normal
*Covariate parameters*					
$\beta_{F,\text{Dose}}$	–	−0.102 (27%)			
*Residual variability*					
Proportional error	–	0.179 (2.1%)			

*Note*: Significant digits: 3. Relative standard error in parentheses.

Abbreviation: $\beta_{F,\mathrm{Dose}}$, covariate coefficient‐effect of dose on F; CL, clearance; CV, coefficient of variation; F, bioavailability; IIV, inter‐individual variability; $Q_{1}$, inter‐compartmental clearance; $T_{k,0}$, duration of 0th‐order absorption; $T_{lag,1}$, absorption lag‐time; $V_{c}$, volume of centrale compartment; $V_{p,1}$, volume of first peripheral compartment.

^a^ IIV values reported in standard deviation.

^b^ CV [%] calculated as $\frac{\mathrm{IIV}}{\text{Value}}\cdot 100$ and $\sqrt{e^{{\mathrm{IIV}}^{2}}-1}\cdot 100$ for normal and log‐normal distributed variables, respectively.

### Blood‐stage model

In field studies with patients presenting with malaria infection, the parasite growth rate cannot be observed before drug administration as is possible in a VIS. Therefore, it was assumed that patients and volunteers have a similar growth rate, $GR_{B}$, which was estimated to be 0.070 per h with no IIV due to high shrinkage. The final blood‐stage PKPD model (Table S1) showed adequate overall diagnostic performance, with parasitemia profiles correctly characterized for all subjects (Text S1, Step 2). The median $MIC_{B}$ of DSM265 was estimated to be 1.6 μg/ml (observed range, 0.7–2.6) and 2.5 μg/ml (observed range, 1.3–3.1) for volunteers and patients, respectively.

### Model growth of hepatic schizonts

Where the blood‐stage equation (Equation 1.2) without hepatocyte transfer function was used to estimate the growth rate of parasites in blood‐stage $GR_{B}$ following liver infection, the growth rate was estimated to be 0.062 per h with an IIV of 0.12 (Table S2; Text S1, Step 3). The fraction of the 3200 inoculated sporozoites that became infectious (invaded hepatocytes) was estimated to be 0.12% with an IIV of 0.14 (Table S3; Text S1, Step 4), corresponding to four infected hepatocytes (90% range, 3–5).

### Model of drug activity against hepatic schizonts

Assuming that the $E_{max}$ for liver was equal to that in blood and that the turnover rate $k_{in}$ was the same for both pools allowed estimation of the liver‐stage $EC_{50,L}$ of 1.6 μg/ml with an IIV of 0.45 (Table 3; Text S1, Step 5), corresponding to a liver‐stage $MIC_{L}$ of 1.5 μg/ml (90% range, 0.7–3.2). It is worth noting that $EC_{50,L}$ and $MIC_{L}$ are expressed as blood concentrations that characterize activity in the liver and not as liver concentrations.

**TABLE 3:** Population parameter estimates of the chemoprophylaxis model for DSM265 in the spz HuCh (Text S1, Step 5)

Parameter	Unit	Value	IIV^a^	CV^b^	Distribution
*Typical parameters*					
spz/bite	p^c^	640^d^	0^d^	0%	Log‐normal
$F_{inc}$	–	0.00119^d^ ^,^ ^g^	0.142^d^ ^,^ ^g^	14.3%	Log‐normal
$GR_{L}$	1/h	0.0716^d^	0.118^d^ ^,^ ^f^	11.8%	Log‐normal
$E_{max,L}$	1/h	0.205^d^ ^,^ ^e^	0^d^	0%	Log‐normal
$EC_{50,L}$	μg/ml	1.7 (3.99%)	0.444 (20.1%)	46.7%	Log‐normal
$h_{L}$	–	10^d^ ^,^ ^e^	0^d^	0%	Log‐normal
$k_{LB}$	1/h	6^d^	0^d^	0%	Log‐normal
$T_{50}$	h	144^d^	0^d^	0%	Log‐normal
$\sigma_{LB}$	h	0.1^d^	0^d^	0%	Log‐normal
$GR_{B}$	1/h	0.0624^d^ ^,^ ^f^	0.118^d^ ^,^ ^f^	11.8%	Log‐normal
$E_{max,B}$	1/h	0.205^d^ ^,^ ^e^	0^d^	0%	Log‐normal
$EC_{50,B}$	μg/ml	1.25^d^ ^,^ ^e^	0.444 (20.1%)	46.7%	Log‐normal
$h_{B}$	–	10^d^ ^,^ ^e^	0^d^	0%	Log‐normal
$k_{in}$	1/h	0.0771^d^ ^,^ ^e^	0^d^	0%	Log‐normal
*Correlation parameters*					
$\rho_{GR_{L},GR_{B}}$	–	1^d^			
$\rho_{EC_{50,L},EC_{50,B}}$	–	1^d^			
*Residual variability*					
Additive error	ln(p/ml)	2.66 (11.7%)			

*Note*: Significant digits: 3. Relative standard error in parentheses.

Abbreviations: $\sigma_{LB}$, time that controls how fast hepatocytes burst around $T_{50}$; B, blood; $E_{max,X}$, maximum killing rate; $EC_{50,X}$, dried blood spot concentration at which half of maximum effect is attained; $F_{inc}$, the fraction of infectious inoculated sporozoites that invade hepatocytes; IIV, inter‐individual variability; $GR_{X}$, growth rate; $h_{X}$, Hill coefficient; $k_{in}$, turnover rate; $k_{LB}$, rate of merozoites invading erythrocytes once they leave hepatocytes; L, liver; spz/bite, sporozoites per bite; $T_{50}$, time at which half of the hepatocytes burst after infection.

^a^ IIV values reported in standard deviation.

^b^ Coefficient of variation [%] calculated as $\frac{\mathrm{IIV}}{\text{Value}}\cdot 100$ and $\sqrt{e^{{\mathrm{IIV}}^{2}}-1}\cdot 100$ for normal and log‐normal distributed variables, respectively.

^c^ Unit p refers to number of parasites.

^d^ Parameter fixed for the final run (i.e. during step 5 – see Text S1).

^e^ Estimated at step 2 (see Text S1 & Table S1).

^f^ Estimated at step 3 (see Text S1 & Table S2).

^g^ Estimated at step 4 (see Text S1 & Table S3).

### Model validation

**FIGURE 3:**
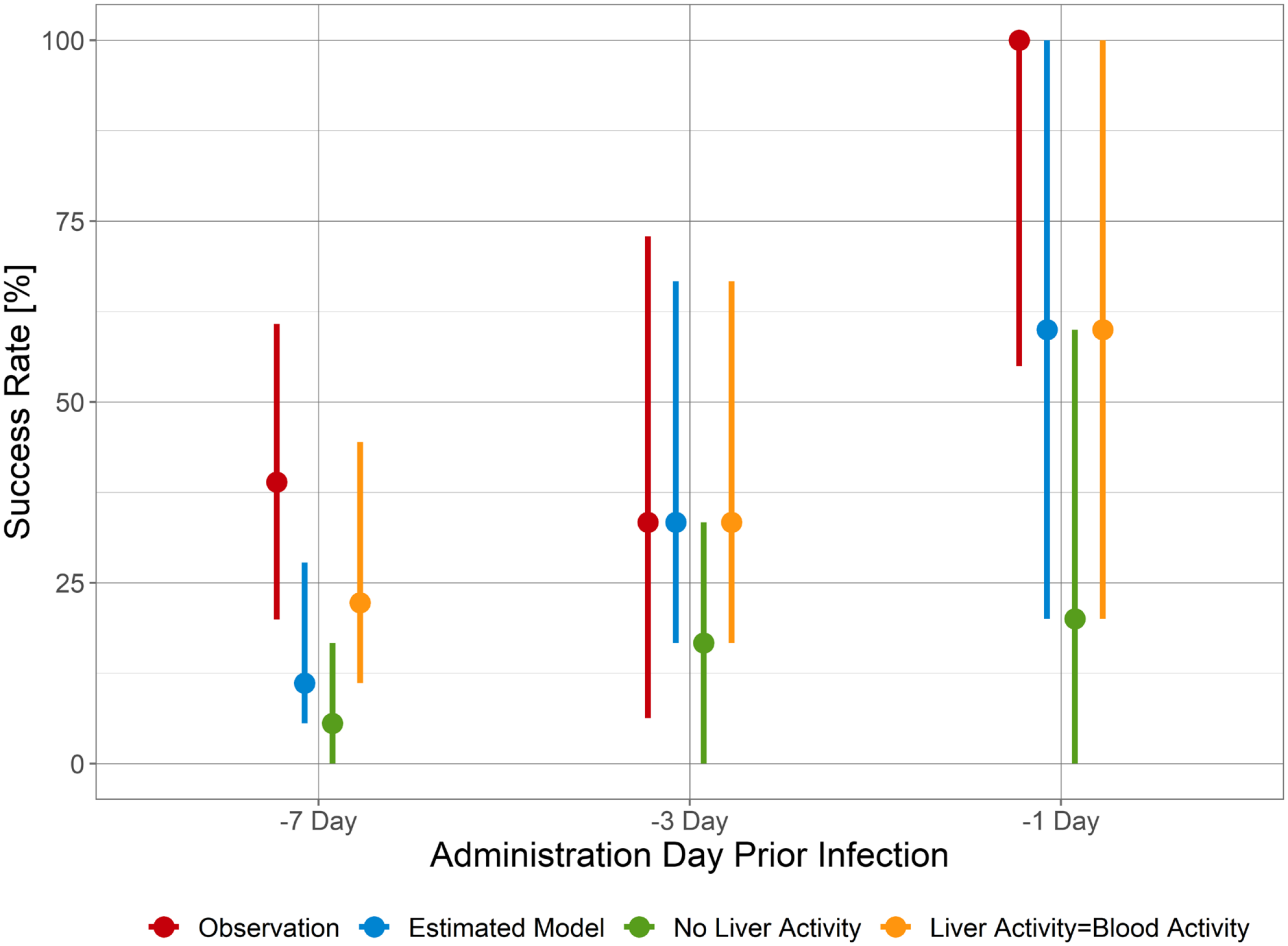
Observed and predicted cure success rates at Day 28 for DSM265 (median and 90% confidence interval): observed in red, predicted from estimated model in blue, predicted assuming no liver activity in green, and predicted assuming liver activity to be identical to blood activity in yellow.

For each treatment group, the observed and predicted success rates were compared to evaluate the predictability of the full PKPD model (Figure 3). The predicted CIs overlap with the observed one for all treatment days. However, due to the small number of subjects, variability was large, especially for Day −1.

### Model assessment

As sporozoites transit the liver prior to being observed in the blood, presystemic antimalarial drug activity will affect observed parasitemia; the unknown is the extent.^16^ Therefore, for each treatment group, success rates were predicted assuming either no liver activity or the same for both the liver and blood stages. When no liver activity was assumed, predictions underestimated the ability of DSM265 to protect patients (Figure 3). However, if liver and blood activities were assumed the same, predictions were closer to observations, albeit with more accurate results for Days −7 and −3 than for Day −1.

### Posology prediction

**FIGURE 4:**
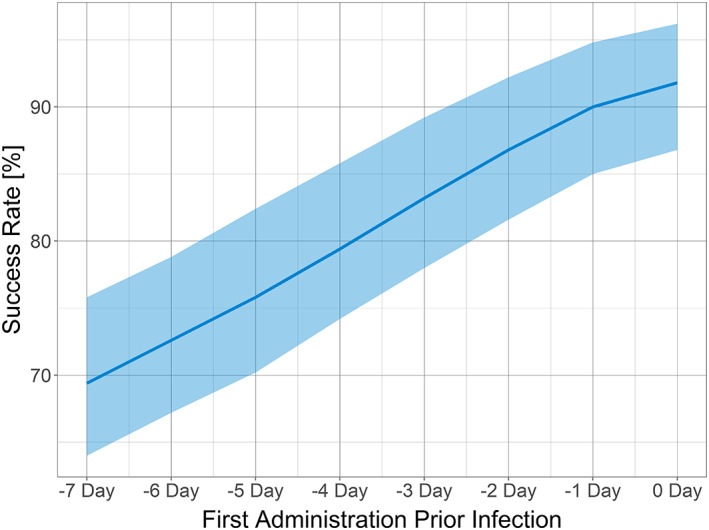
Effect of varying the day of the first 400 mg dose of DSM265 prior to sporozoite infection (Day 0) followed by a second dose 7 days later on the success rate for preventing malaria parasitemia at 28 days postinfection. The solid line represents the median, and the shaded area represents the 90% confidence interval.

DSM265 protection was evaluated in eight simplified real‐life scenarios, where infection occurred on Day 0 with two 400‐mg doses of DSM265 administered weekly, with the first dose administered between Day −7 and Day 0 prior to infection. The model predicted that the closer to infection the first administration occurred, the better a subject would be protected (Figure 4). This could be explained by the second dose extending the duration of DSM265 exposure, thereby adding greater blood‐stage protection to that afforded in the liver. Considering the long terminal half‐life of 100 h and the estimated MIC of 1.5 μg/ml for the liver activity of DSM265, it may be considered surprising that individuals infected the furthest from the first drug administration were not fully protected. However, MIC is the concentration such that killing equals growth; it is not parasiticidal. For a patient to be malaria free, one needs far higher concentrations. Furthermore, there is IIV in the PK and PD parameters such that some patients will have too short a half‐life, too large a volume, and/or too high a MIC; these will be the failures.

## DISCUSSION

A chemoprophylaxis PKPD model with two compartments to mathematically represent liver‐ and blood‐stage parasitemia was created to support the development of chemoprophylactic drugs. However, because liver‐stage parasites cannot be directly observed, there remained the challenge of estimating parasite transfer and drug‐induced killing parameters. By assuming similar activity for DSM265 for both the liver and blood stages, as observed in vitro,^14^ together with knowledge of parasite dynamics from two different designs of clinical study within a semimechanistic mathematical model, it was possible to estimate liver‐stage growth and initial infection level. By integrating spz HuCh data, a final PKPD model was constructed that had the ability to describe the level of chemoprotection delivered by DSM265 and therefore predict clinical outcomes. Furthermore, when only clinical blood‐stage activity is known, assuming similar characteristics between liver‐ and blood‐stage activities could be used to assess the levels of chemoprotection afforded by compounds with similar in vitro liver‐ and blood‐stage activities through simulations. For compounds with different in vitro liver‐ and blood‐stage activities, one could assess the level of chemoprotection by correcting $EC_{50,L}$, with the in vitro ratio between liver‐ and blood‐stage activities and difference in tissue binding (liver vs. blood/plasma). This may be useful in selecting candidate compounds for phase Ib (spz HuCh studies) and phase II clinical trials. The proposed scenarios described herein could be used to help select suitable doses and regimens to achieve the required success rates for phase II. However, to get closer to phase II study designs run under real‐world conditions, further factors need consideration. These include different infection rates in different geographic areas and different levels of infection, treatment durations, and compliance. This could be achieved by combining knowledge from epidemiology and “adherence behavior”. Future model development could also be adapted to include multiple mosquito bite scenarios, incorporating the unpredictability of real‐world conditions. Such a model could be used to estimate what drug dose would be required to produce higher rates of protection, for instance, in 75%–95% of cases, or to predict whether practical frequencies, such as monthly, could be viable.

It was also necessary to challenge the assumptions of the model. This was done by simulating spz HuCh assuming no liver activity or similar levels of activity for the liver and blood. This showed that liver activity cannot be ignored for DSM265, but imputing blood activity into liver activity already gave feedback on the level of chemoprotection afforded.

One could also use sensitivity analysis to quantify the effect of various assumptions, such as duration of liver infection $T_{50}$, its growth rate $GR_{L}$, distribution of merozoite release (controlled by $\sigma_{LB}$), differences between stages in killing rate ($E_{max,L}$ vs. $E_{max,B}$), or in in vitro potency ($EC_{50,L}$ vs. $EC_{50,B}$), their impact on estimation of liver‐stage PD parameters, and clinical outcomes. Due to the low numbers of parasites at the beginning of infection, parasite dynamics should be better described by a randomly varying stochastic model than a continuous model. However, although median parasite dynamic might still be well described, variability is more difficult to interpret as it is not straightforward to fix a threshold for cure (a parasite reduction threshold below that which can claim complete cure). This could explain why the model underpredicted the efficacy of DSM265 when administered 1 day prior to infection.

In conclusion, one of the biggest challenges facing malaria prevention is a problem of knowledge concerning liver activity during the malaria parasite infection process. However, the ability to infer such information by way of mathematical modeling is a means of changing this. If blood‐stage activity is known, then PKPD models can be used to assess the levels of chemoprotection delivered by a new compound. If liver‐stage activity were characterized in in vitro assays, one can refine those assessments, as explained previously. By combining with sensitivity analysis to derisk uncertainty on liver parameters, this could be useful in selecting suitable compounds for phase Ib and II clinical trials, especially if combined with knowledge from epidemiology and patient compliance. This would augment the transition from preclinical through translational medicine to later confirmatory stages of development for new and improved antimalarials.

## AUTHOR CONTRIBUTIONS

M.H.C.‐R. wrote the manuscript. M.H.C.‐R., N.A., N.G., and J.J.M. designed the research. M.H.C.‐R. performed the research. M.H.C.‐R., N.A., L.B., O.F.E., R.F., C.F., M.G., and J.W. analyzed the data. M.H.C.‐R., N.A., L.B., O.F.E., R.F., C.F., M.G., and J.W. contributed to the new analytical tools.

## FUNDING INFORMATION

This work was funded in whole by Medicines for Malaria Venture (MMV). A full list of donors to MMV is available on its website (www.mmv.org) and in its annual report. MMV receives support from Bill and Melinda Gates Foundation Grant INV‐007155.

## CONFLICT OF INTEREST

M.H.C.‐R., J.J.M., and N.G. are employees of Medicines for Malaria Venture, as was N.A. All other authors declared no competing interests for this work.

## Supporting information


Table S1

Table S2

Table S3

Text S1

Data S1

Data S2

Data S3

Data S4

Data S5
