# Supplementary material for: A pharmacokinetic–pharmacodynamic model for chemoprotective agents against malaria
Source: CPT Pharmacometrics Syst Pharmacol. 2022 Nov 22;12(1):50–61. doi: 10.1002/psp4.12875 (PMC9835136; doi:10.1002/psp4.12875)
Supplement: Supplementary file 1 — Table S1 [file PSP4-12-50-s004.pdf]

Table S1 **Population parameter estimates of the final model describing DSM265 blood-stage activity in volunteers and patients with uncomplicated *P. falciparum* malaria model based on the IBSM (9) and Phase IIa (12) studies (see Text S1 – Step 2).**

PARAMETER	UNIT	VALUE	RSE	SHRINKAGE
**Typical parameters**				
$\ln\left(P_B(t=0)\right)$	ln(p/mL)	14.6	2.05%	-
GR_B	1/hr	0.0703	4.29%	-
$E_{max,B}$	1/hr	0.205	3.38%	-
$EC_{50,B}$	ug/mL	1.25	16.1%	-
$Hill_B$	-	10 (FIX)	-	-
k_{in}	1/hr	0.0771	13.9%	-
**Inter-individual variability**				
$\omega_{\ln(P_B(t=0))}$	-	1.38	7.51%	3.4%
ω_{GR_B}	-	0 (FIX)	-	-
$\omega_{E_{max,B}}$	-	0.265	15.8%	6.6%
$\omega_{EC_{50,B}}$	-	0.552	21%	40%
ω_{Hill_B}	-	0 (FIX)	-	-
$\omega_{k_{in}}$	-	0 (FIX)	-	-
**Parameter-Covariate relationships**				
$\beta_{\ln(P_B(t=0)),STUDYTYPEN_2}$	-	-12	3.72%	
**Residual Variability**				
$error_{ADD1}$	ln(p/mL)	1.13	2.66%	
Objective function		2083		
AIC		2103		
BIC		2119		

Significant digits: 3 (Objective function rounded to closest integer value)
Omega values reported as standard deviation.
