# Supplementary material for: A pharmacokinetic–pharmacodynamic model for chemoprotective agents against malaria
Source: CPT Pharmacometrics Syst Pharmacol. 2022 Nov 22;12(1):50–61. doi: 10.1002/psp4.12875 (PMC9835136; doi:10.1002/psp4.12875)
Supplement: Supplementary file 2 — Table S2 [file PSP4-12-50-s006.pdf]

Table S2 **Population parameter estimates of the final model describing *P. falciparum* growth during blood-stage following liver-stage infection in volunteers based on the placebo volunteers in the spz HuCh studies (10,11) and the published studies in Coffeng *et al.* 2017 (13) (see Text S1 – Step 2).**

PARAMETER	UNIT	VALUE	RSE	SHRINKAGE
**Typical parameters**				
$\ln\left(P_B(t=0)\right)$ [a]	ln(p/mL)	-5.98	5.32%	-
GR_B	1/hr	0.0624	3.05%	-
**Inter-individual variability**				
ω_{GR_B}	-	0.118	10.3%	6.2%
$\omega_{\ln(P_B(t=0))}$	-	0 (FIX)	-	-
**Residual Variability**				
$error_{ADD1}$	ln(p/mL)	1.39	3.69%	-
Objective function		1917	-	-
AIC		1925	-	-
BIC		1934	-	-

Significant digits: 3 (Objective function rounded to closest integer value)

Omega values reported as standard deviation.

[a] Parameter for fit purpose as the parasite is not in the blood at $t = 0$ (*i.e.* no biological meaning). The goal was to estimate GR_B.
