# Supplementary material for: A pharmacokinetic–pharmacodynamic model for chemoprotective agents against malaria
Source: CPT Pharmacometrics Syst Pharmacol. 2022 Nov 22;12(1):50–61. doi: 10.1002/psp4.12875 (PMC9835136; doi:10.1002/psp4.12875)
Supplement: Supplementary file 3 — Table S3 [file PSP4-12-50-s005.pdf]

Table S3 **Population parameter estimates of the final model describing *P. falciparum* growth during liver-stage following sporozoite inoculum based on the placebo volunteers in the spz HuCh studies (10,11) and the published studies in Coffeng *et al.* 2017 (13) (see Text S1 – Step 3).**

PARAMETER	UNIT	VALUE	RSE	SHRINKAGE
**Typical parameters**				
SPZ/Bite	p	640 (FIX)	-	-
F_{inc}	-	0.00119	6.03%	-
GR_L	1/hr	0.0716 (FIX)	-	-
k_{LB}	1/hr	6 (FIX)	-	-
T_{50}	hr	144 (FIX)	-	-
σ_{LB}	hr	0.1 (FIX)	-	-
**Inter-individual variability**				
$\omega_{\mathrm{SPZ/Bite}}$	-	0 (FIX)	-	-
$\omega_{F_{inc}}$	-	0.142	126%	75%
ω_{GR_L}	-	0 (FIX)	-	-
$\omega_{k_{LB}}$	-	0 (FIX)	-	-
$\omega_{T_{50}}$	-	0 (FIX)	-	-
$\omega_{\sigma_{LB}}$	-	0 (FIX)	-	-
**Residual Variability**				
$error_{ADD1}$	ln(p/mL)	1.32	3.11%	
Objective function		1499		
AIC		1505		
BIC		1512		

Significant digits: 3 (Objective function rounded to closest integer value)
Omega values reported in standard deviation.
