# Supplementary material for: A pharmacokinetic–pharmacodynamic model for chemoprotective agents against malaria
Source: CPT Pharmacometrics Syst Pharmacol. 2022 Nov 22;12(1):50–61. doi: 10.1002/psp4.12875 (PMC9835136; doi:10.1002/psp4.12875)
Supplement: Supplementary file 4 — Text S1 [file PSP4-12-50-s002.pdf]

Text S1: Goodness-of-fit diagnostic plots.

Cherkaoui-Rbati *et al.* "A PKPD Model for Chemoprotective Agents against Malaria."

Individual subjects' data and predictions by analysis step and study

The following plots show each individual subject's data, individual and population predicted model curves for the pharmacokinetics of DSM265 and pharmacodynamics of *Plasmodium falciparum* blood counts. Solid lines are the individual, dotted are population predictions. Colours distinguish individuals.

Samples analysing below the limit of quantification (BLOQ) are plotted separately from above LOQ data. The number of samples analysing BLOQ within each time bin are plotted as a proportion of the total number of samples within the bin. Overlaid are the individual predictions as blue lines classified as being BLOQ as a proportion of the total. The blue bands are the 95% confidence intervals calculated using a binomial confidence interval by the exact method as per `library(binom)` in `R`. Time was binned for each step and study using the function `auto_bin()` from `library(vpc)`.

Analysis Step 1 Pharmacokinetics, individuals' data and predictions

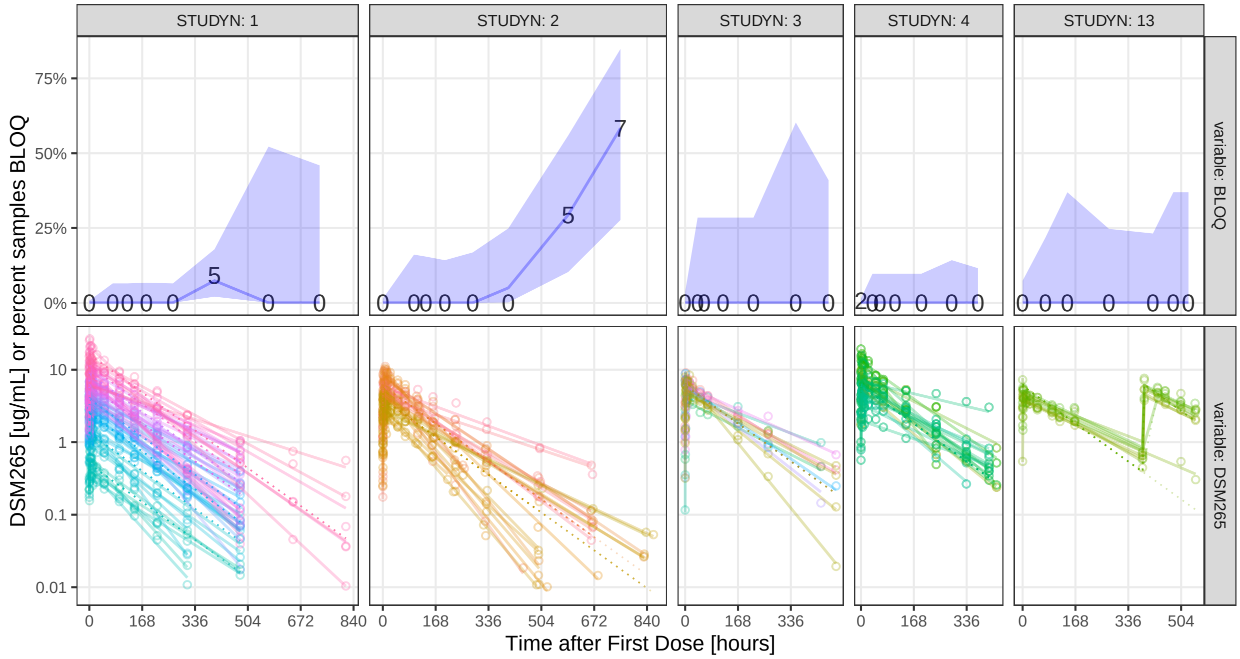

Figure 1: The first row of plots shows the percent of predicted PK samples BLOQ after DSM265 administration (solid line) with its 95% confidence interval (blue band) compared to the observations indicating the percent of samples BLOQ (ordinate) and the number of samples BLOQ (label). The second row of plots shows the individual (solid line) and population (dotted line) DSM265 PK prediction compared to the observations (open circle).

Analysis Step 2 Blood stage estimation, individuals' data and predictions

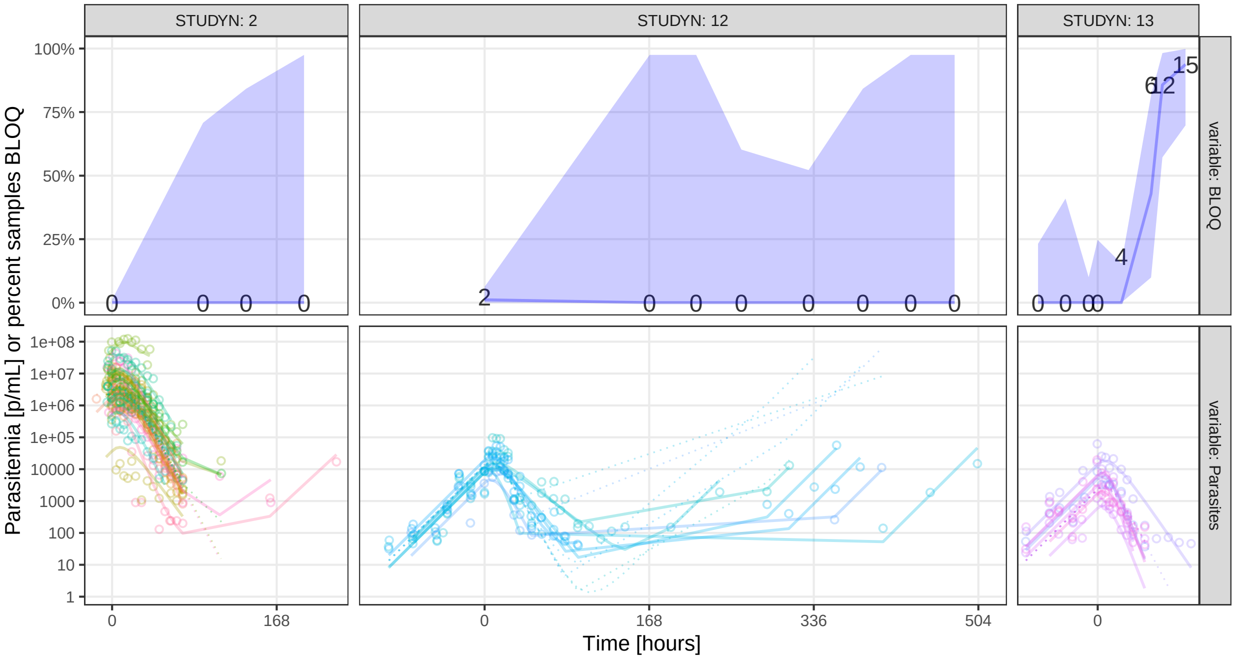

Figure 2: The first row of plots shows the percent of predicted parasitemia samples BLOQ after DSM265 administration for treatment (solid line) with its 95% confidence interval (blue band) compared to the observations indicating the percent of samples BLOQ (ordinate) and the number of samples BLOQ (label). The second row of plots shows the individual (solid line) and population (dotted line) parasitemia prediction compared to the observations (open circle).

Analysis Step 3 Liver stage, blood growth estimation, individuals' data and predictions

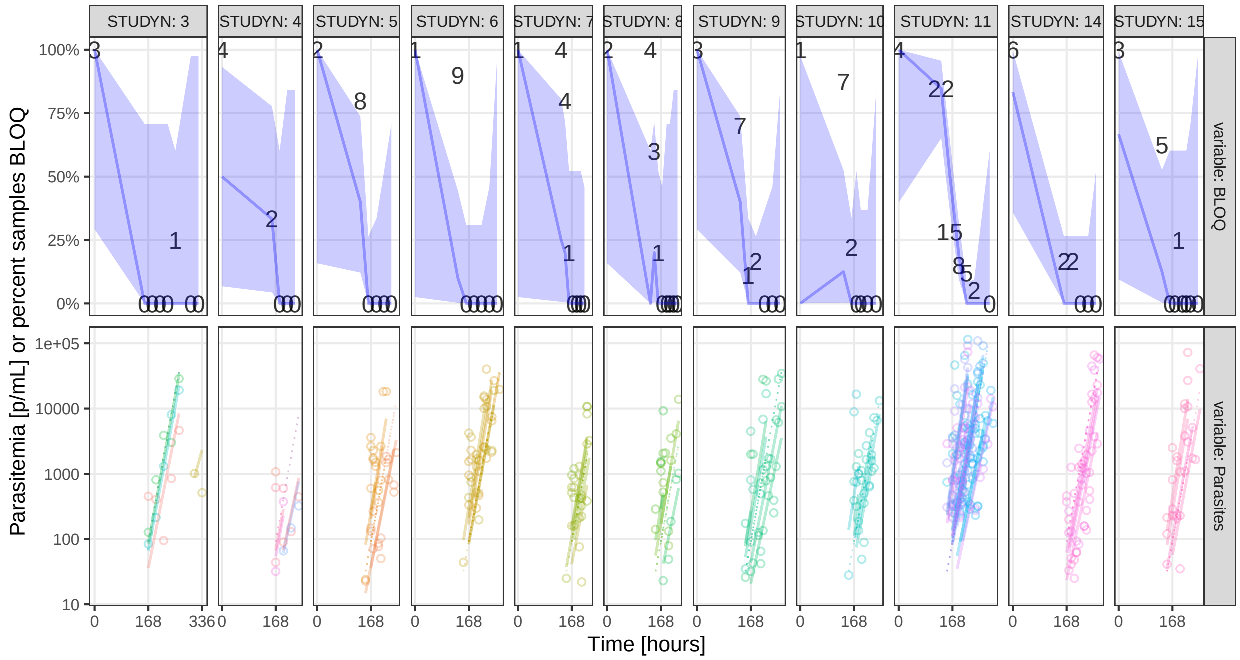

Figure 3: The first row of plots shows the percent of predicted parasitemia samples BLOQ in placebo after liver infection (solid line) with its 95% confidence interval (blue band) compared to the observations indicating the percent of samples BLOQ (ordinate) and the number of samples BLOQ (label). The second row of plots shows the individual (solid line) and population (dotted line) parasitemia prediction compared to the observations (open circle).

Analysis Step 4 Liver stage, F.inv estimation, individuals' data and predictions

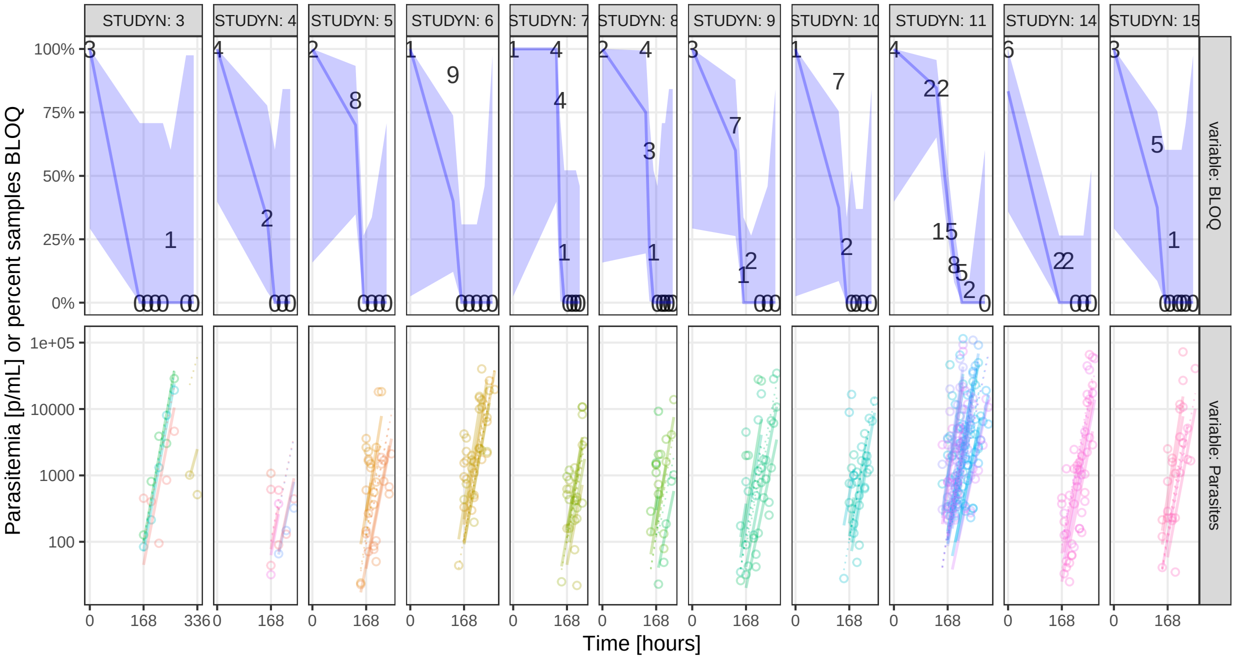

Figure 4: The first row of plots shows the percent of predicted parasitemia samples BLOQ in placebo after liver infection (solid line) with its 95% confidence interval (blue band) compared to the observations indicating the percent of samples BLOQ (ordinate) and the number of samples BLOQ (label). The second row of plots shows the individual (solid line) and population (dotted line) parasitemia prediction compared to the observations (open circle).

Analysis Step 5 Liver stage, EC50 estimation, individuals' data and predictions

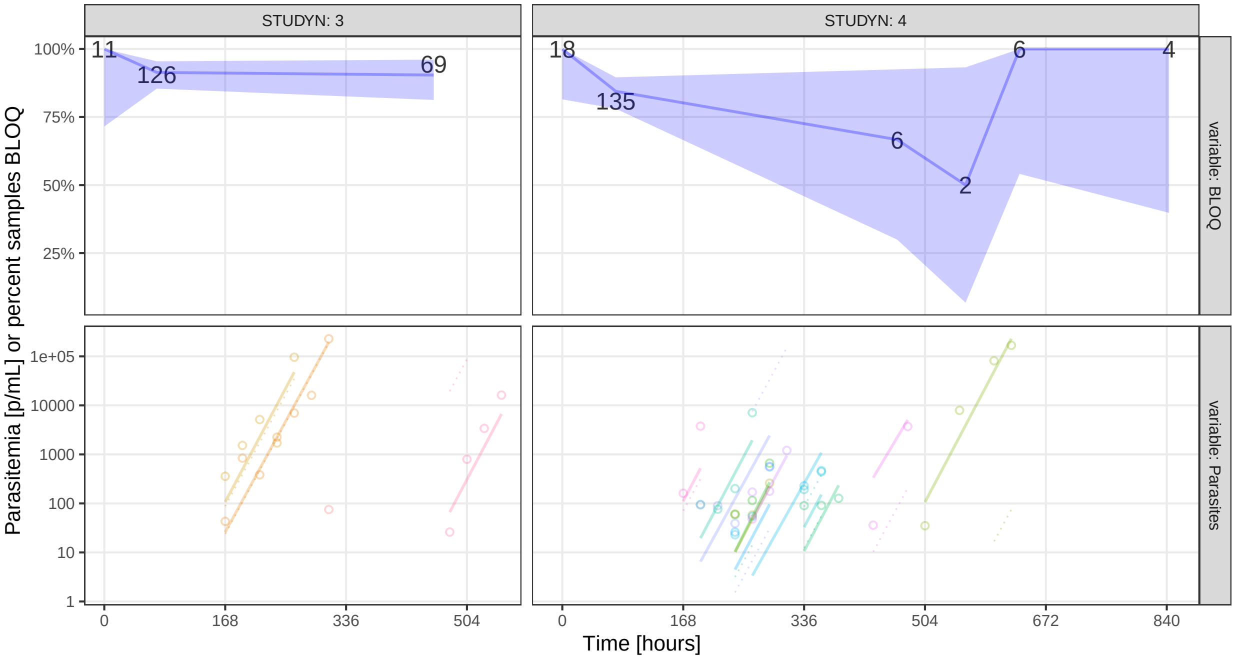

Figure 5: The first row of plots shows the percent of predicted parasitemia samples BLOQ after DSM265 administration as a chemoprophylaxis treatment (solid line) with its 95% confidence interval (blue band) compared to the observations indicating the percent of samples BLOQ (ordinate) and the number of samples BLOQ (label). The second row of plots shows the individual (solid line) and population (dotted line) parasitemia prediction compared to the observations (open circle).

Weighted residuals and BLOQ accuracy of prediction

The following are time-based plots by analysis step and study number for:

i) The number of samples analysing BLOQ within each time bin are plotted as a proportion of the total number of samples. Overlaid are the individual predictions as blue lines classified as being BLOQ as a proportion of the total. The blue bands are 95% confidence intervals calculated using a binomial confidence interval by the exact method as per `library(binom)` in `R`. Time was binned for each step and study using the function `auto_bin()` from `library(vpc)`.

ii) Individual weighted residuals for the data above the limit of quantification.

iii) Population weighted residuals for the data above the limit of quantification.

Analysis Step 1 Pharmacokinetics residuals and BLOQ accuracy of prediction

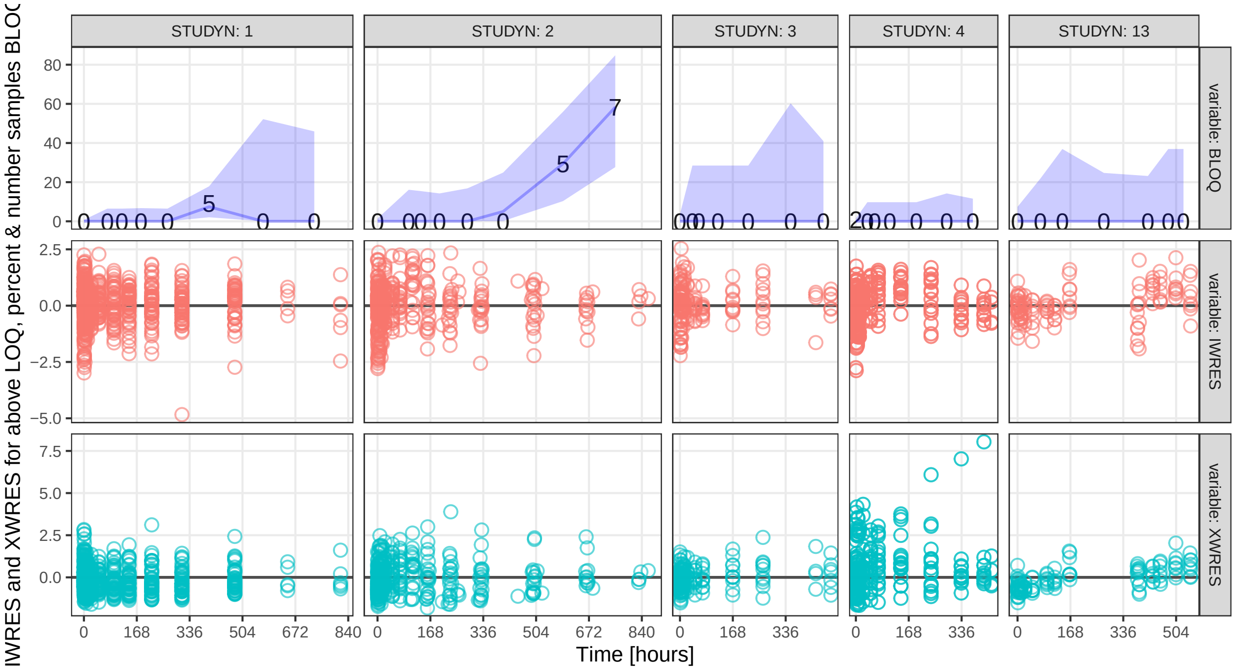

Figure 6: The first row of plots shows the percent of predicted PK samples BLOQ after DSM265 administration (solid line) with its 95% confidence interval (blue band) compared to the observations indicating the percent of samples BLOQ (ordinate) and the number of samples BLOQ (label). The second and third rows of plots show the individual and population weighted residual, respectively.

Analysis Step 2 Blood stage estimation residuals and BLOQ accuracy of prediction

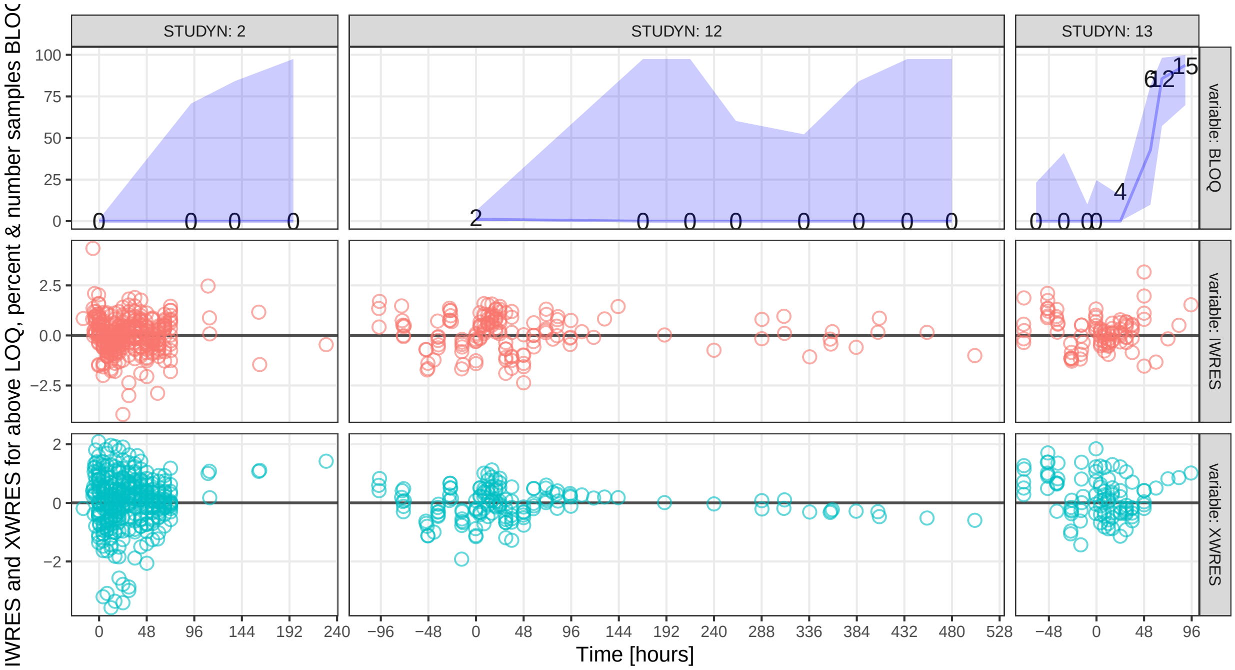

Figure 7: The first row of plots shows the percent of predicted parasitemia samples BLOQ after DSM265 administration for treatment (solid line) with its 95% confidence interval (blue band) compared to the observations indicating the percent of samples BLOQ (ordinate) and the number of samples BLOQ (label). The second and third rows of plots show the individual and population weighted residual, respectively.

Analysis Step 3 Liver stage blood growth estimation residuals and BLOQ accuracy of prediction

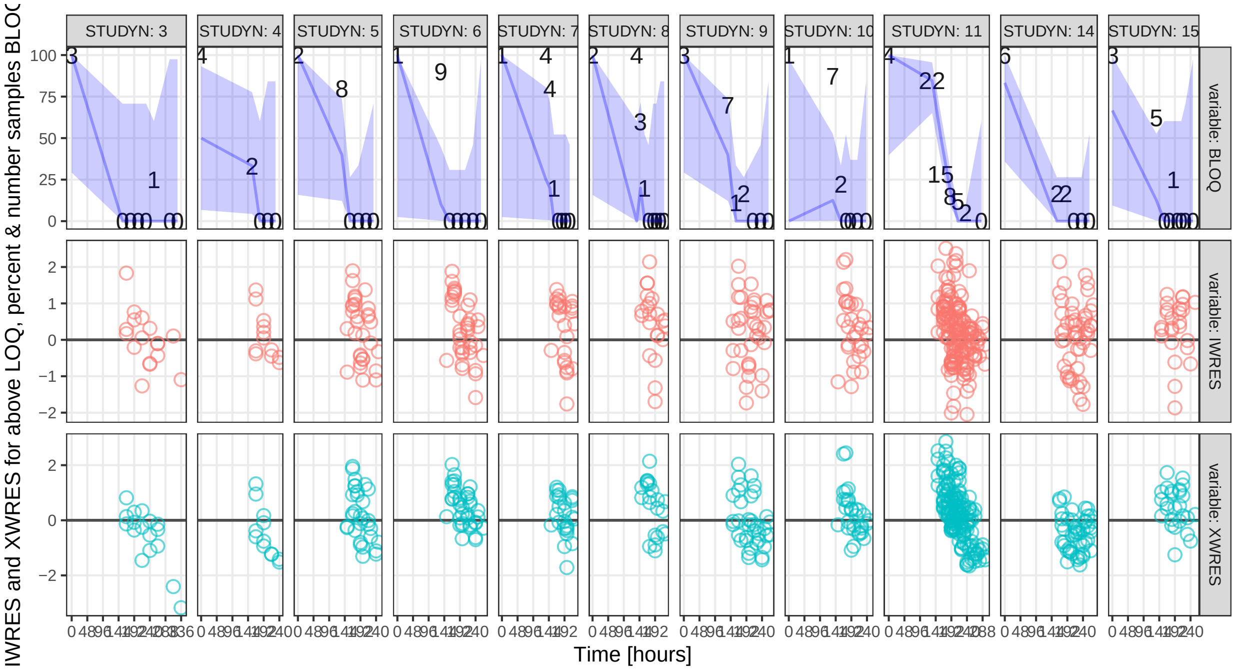

Figure 8: The first row of plots shows the percent of predicted parasitemia samples BLOQ in placebo after liver infection (solid line) with its 95% confidence interval (blue band) compared to the observations indicating the percent of samples BLOQ (ordinate) and the number of samples BLOQ (label). The second and third rows of plots show the individual and population weighted residual, respectively.

Analysis Step 4 Liver stage F.inv estimation residuals and BLOQ accuracy of prediction

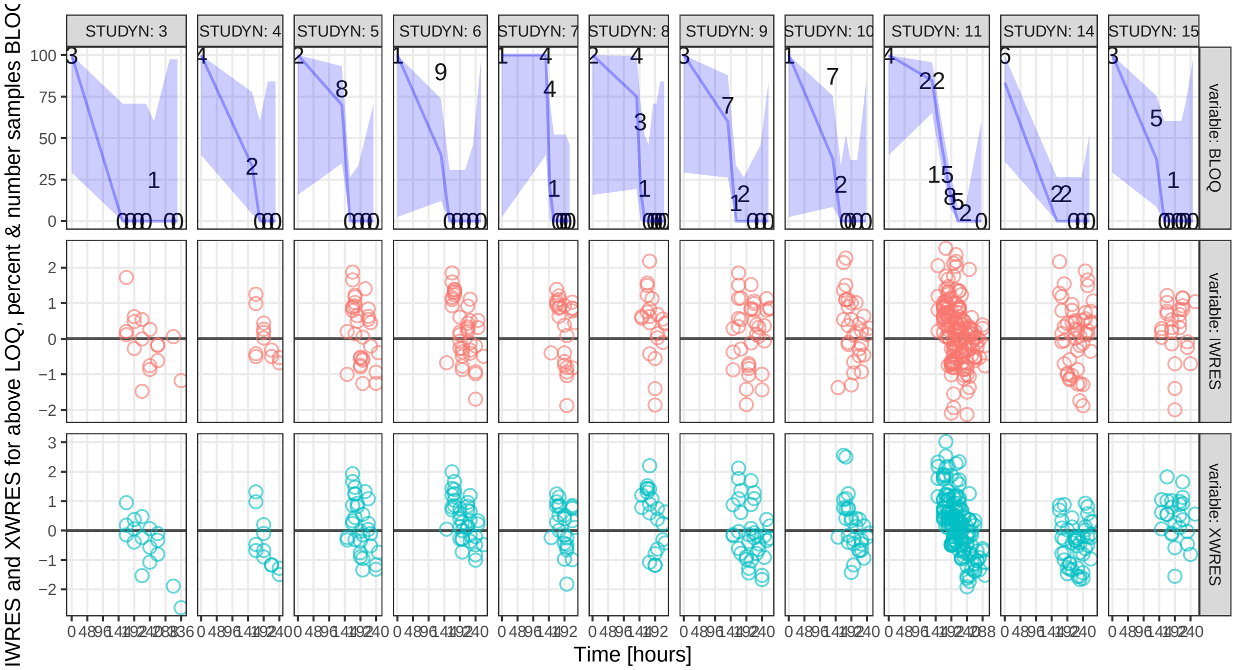

Figure 9: The first row of plots shows the percent of predicted parasitemia samples BLOQ in placebo after liver infection (solid line) with its 95% confidence interval (blue band) compared to the observations indicating the percent of samples BLOQ (ordinate) and the number of samples BLOQ (label). The second and third rows of plots show the individual and population weighted residual, respectively.

Analysis Step 5 Liver stage EC50 estimation residuals and BLOQ accuracy of prediction

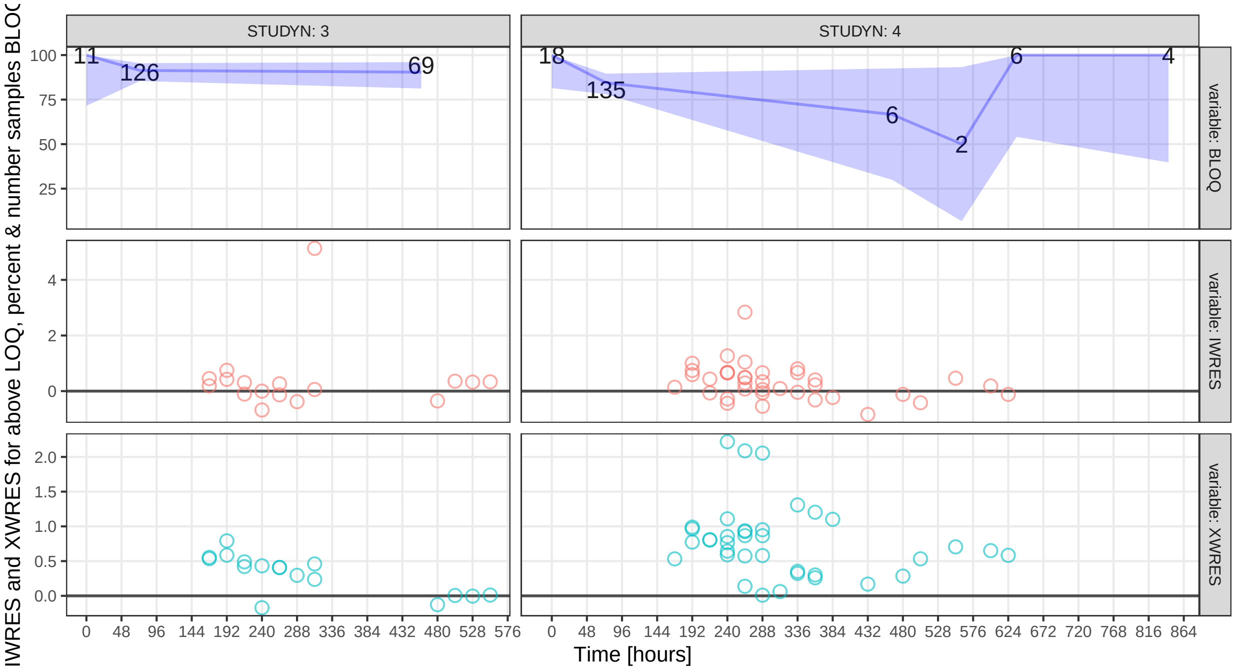

Figure 10: The first row of plots shows the percent of predicted parasitemia samples BLOQ after DSM265 administration as a chemoprophylaxis treatment (solid line) with its 95% confidence interval (blue band) compared to the observations indicating the percent of samples BLOQ (ordinate) and the number of samples BLOQ (label). The second and third rows of plots show the individual and population weighted residual, respectively.

Density plots for bias and normality of distributions of above LOQ residuals

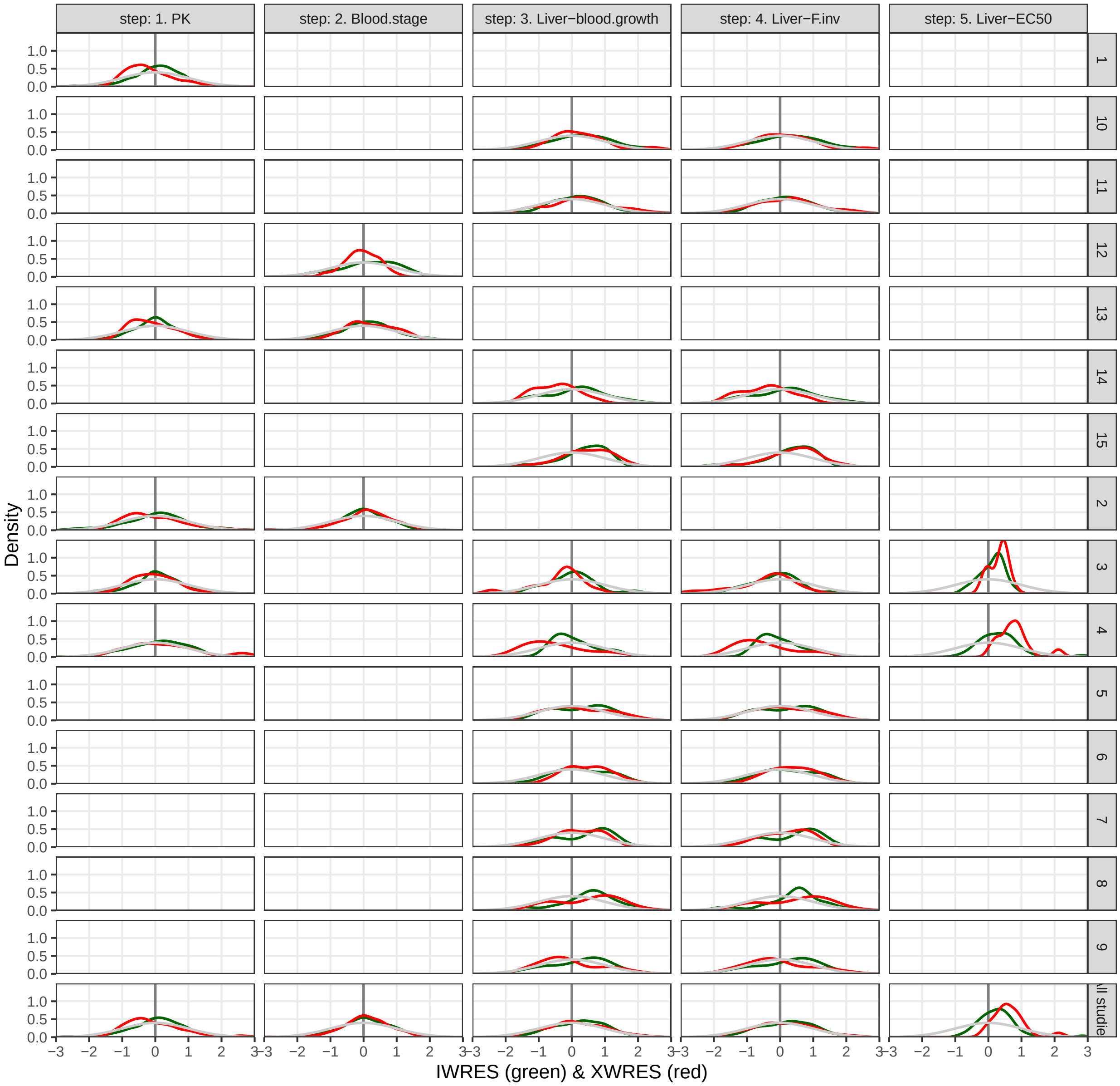

Figure 11: Density plots for the data above the limit of quantification based upon the individual weighted residuals (green) and population weighted residuals (red) compared to the standard normal distribution (gray).

Philip Lowe, It's all in the dose Ltd, 28-août-2022
